# Microglial glutaminase 1 mediates chronic restraint stress-induced depression-like behaviors and synaptic damages

**DOI:** 10.1038/s41392-023-01699-8

**Published:** 2023-12-15

**Authors:** Huili Chen, Shengyang Fu, Xiangyu Li, Meng Shi, Jiazhen Qian, Shu Zhao, Ping Yuan, Lu Ding, Xiaohuan Xia, Jialin C. Zheng

**Affiliations:** 1 Center for Translational Neurodegeneration and Regenerative Therapy, Tongji Hospital affiliated to Tongji University School of Medicine, 200065 Shanghai, China; 2 Department of Cardio-Pulmonary Circulation, Shanghai Pulmonary Hospital, School of Medicine, Tongji University, Shanghai, China; 3 Translational Research Institute of Brain and Brain-Like Intelligence, Shanghai Fourth People’s Hospital affiliated to Tongji University School of Medicine, 200434 Shanghai, China

**Keywords:** Neuroimmunology, Diseases of the nervous system

**Dear Editor**,

Major depressive disorder (MDD) is one of the most common psychiatric illnesses that significantly increase the risk of suicide.^1^ Stress-triggered dysfunctions of microglia have been identified as a commonly occurred pathological feature of MDD.^1–3^ Microglial dysfunction contributes to the pathogenesis of MDD via immunoresponses/neuroinflammation-mediated neural damage and pathological synapse loss-mediated neural circuit disruption.^1^ Although the involvement of microglia in MDD has been widely investigated, the molecular mechanisms underlying microglial dysfunction remain largely unknown. Recently, we identified glutaminase 1 (Gls1) as one key protein that participates in microglial dysfunction.^4–6^ Gls1 catalyzes the hydrolysis of glutamine to produce glutamate in the brain.^4^ Besides its well-known role in excitatory neurotoxicity, we found Gls1 up-regulation in microglia in animal models of Alzheimer’s disease and ischemic stroke.^4,7^ Gls1 activates microglia to overproduce cytokines and release inflammatory extracellular vesicles, therefore leading to neuroinflammation in animal models of Alzheimer’s disease and ischemic stroke.^4–6^ More importantly, Gls1 has been found to be up-regulated in the brains of MDD patients, and microglial Gls1 deficiency mitigated LPS-induced depression-like behaviors.^8^ However, LPS exposure is not an appropriate model to mimic MDD phenotypes, leaving the involvement of Gls1 in MDD an undetermined question.

To investigate the role of Gls1 in depression, we first established a well-recognized depression-like model that exposed male mice to 2 weeks of chronic restraint stress (CRS).^9^ CRS mice were more immobile in the forced swimming test (FST) and tail suspended test (TST), and less desired to sucrose solution in the sucrose preference test (SPT), displaying typical depression-like behaviors (Supplementary Fig. 1). Depression-like behaviors of CRS mice were still observed 6 weeks post CRS stimulation in another cohort of mice, suggesting long-term effects of CRS exposure on the behavioral abnormality of animals (Supplementary Fig. 2). Both Gls1 transcript and protein levels were significantly increased in the hippocampal (Supplementary Fig. 3a–c) and cortical tissues (Supplementary Fig. 3d–f) of CRS mice, compared with the control ones. Interestingly, elevated expression levels of *Gls1* mRNA and Gls1 protein were observed in CD11b^+^ microglia, but not Glast^+^ astrocytes, or CD11b^−^/Glast^−^ neurons in the hippocampal and cortical tissues (Fig. 1a–c). Immunohistochemical analysis also showed a significant increase in proportions of Gls1^+^ cells in Iba1^+^ cells, but not in GFAP^+^ or Map2^+^ cells, versus controls (Supplementary Fig. 4). The increase of microglial Gls1 protein levels could even be detected in mouse brains 6 weeks after CRS treatment (Supplementary Fig. 5). Although we cannot exclude the possibility of Iba1^+^ macrophage infiltration due to chronic stress conditions, the vast majority of Iba1^+^ cells represent microglia. Therefore, our results clearly indicate increase of microglial Gls1 in CRS mouse brains.

**Fig. 1:**
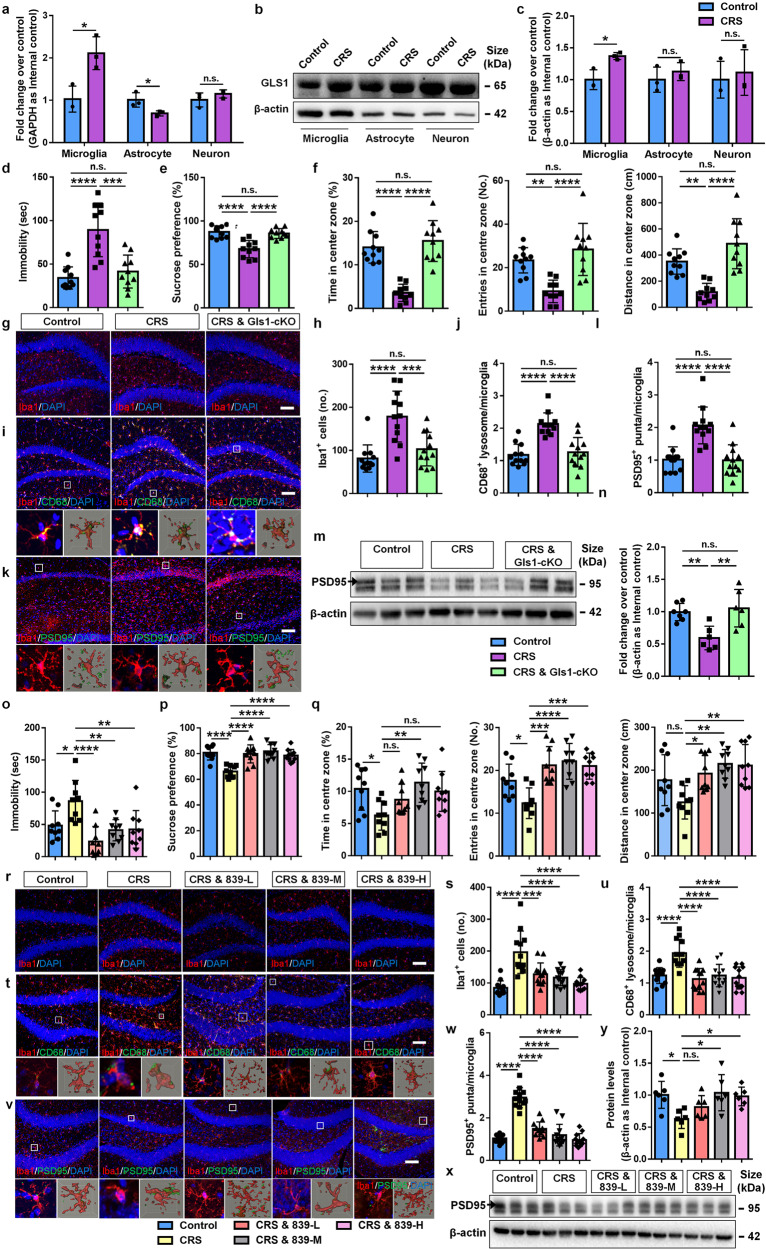
Microglial glutaminase 1 mediates chronic restraint stress-induced depression-like behaviors and synaptic damages. **a** qRT-PCR analysis for *Gls1* mRNA expression in microglia, astrocytes, and neurons (*n* = 3, each dot indicates the sorted cells from 10 animals, unpaired *t*-test). **b** Representative blots for Gls1 protein expression in microglia, astrocytes, and neurons. **c** Quantification results of western blotting analysis (*n* = 3, each dot indicates the sorted cells from 10 animals, unpaired *t-*test). **d** Performance of control, CRS, and Gls1-cKO CRS mice in TST (*n* = 10 animals, one-way ANOVA). **e** Performance of control, CRS, and Gls1-cKO CRS mice in SPT (*n* = 10, one-way ANOVA). **f** Performance of control, CRS, and Gls1-cKO CRS mice in OFT (*n* = 10 animals, one-way ANOVA). **g** Representative images of Iba1 immunostaining (red) of microglia in the dorsal hippocampi of control, CRS, and Gls1-cKO CRS mice. **h** Quantification of Iba1^+^ cell numbers (*n* = 12 slides, 2 slides/animal, one-way ANOVA). **i** Representative images of Iba1 immunostaining (red) and CD68 immunostaining (green) of microglia in the dorsal hippocampi of control, CRS, and Gls1-cKO CRS mice. Magnification and 3D reconstructed images were placed below each panel. **j** Quantification of CD68^+^ lysosome numbers in Iba1^+^ cells *n* = 12 slides, 2 slides/animal, one-way ANOVA). **k** Representative images of Iba1 immunostaining (red) and Psd95 immunostaining (green) of microglia in the dorsal hippocampi of control, CRS, and Gls1-cKO CRS mice. Magnification and 3D reconstructed images were placed below each panel. **l** Quantification of Psd95^+^ puncta numbers in Iba1^+^ cells (*n* = 12 slides, 2 slides/animal, one-way ANOVA). **m** Representative blots for Psd95 protein expression in the hippocampi of control, CRS, and Gls1-cKO CRS mice. **n** Quantification results of western blotting analysis (*n* = 6 animals, one-way ANOVA). **o** Performance of control, CRS, and different doses (L: 3 mg/kg, M: 10 mg/kg, and H: 30 mg/kg) of CB839-treated CRS mice in TST (*n* = 9 animals, one-way ANOVA). **p** Performance of control, CRS, and different doses of CB839-treated CRS mice in SPT (*n* = 9 animals, one-way ANOVA). **q** Performance of control, CRS, and different doses of CB839-treated CRS mice in OFT (*n* = 9 animals, one-way ANOVA). **r** Representative images of Iba1 immunostaining (red) of microglia in the hippocampi of control, CRS, and different doses of CB839-treated CRS mice. **s** Quantification of Iba1^+^ cell numbers (*n* = 12 slides, 2 slides/animal, one-way ANOVA). **t** Representative images of Iba1 immunostaining (red) and CD68 immunostaining (green) of microglia in the dorsal hippocampi of control, CRS, and different doses of CB839-treated CRS mice. Magnification and 3D reconstructed images were placed below each panel. **u** Quantification of CD68^+^ lysosome numbers in Iba1^+^ cells (*n* = 12 slides, 2 slides/animal, one-way ANOVA). **v** Representative images of Iba1 immunostaining (red) and Psd95 immunostaining (green) of microglia in the dorsal hippocampi of control, CRS, and different doses of CB839-treated CRS mice. Magnification and 3D reconstructed images were placed below each panel. **w** Quantification of Psd95^+^ puncta numbers in Iba1^+^ cells (*n* = 12 slides, 2 slides/animal, one-way ANOVA). **x** Representative blots for Psd95 protein expression in the hippocampi of control, CRS, and different doses of CB839-treated CRS mice. **y** Quantification results of western blotting analysis (*n* = 6 animals, one-way ANOVA). Scale Bar: 100 μm (**g**, **i**, **k**, **r**, **t**, **v**). All data are represented as means ± s.d. ****p < 0.0001, ***p < 0.001, **p < 0.01, and *p < 0.05. n.s. non-statistical differences

To examine the contribution of microglial Gls1 to depression, we established microglia-specific conditional Gls1 knockout (CSF1R^creER^ × Gls1-loxp) mice (Gls1-cKO mice) (Supplementary Fig. 6). The protein level of the microglial Gls1 of Gls1-cKO mice was significantly reduced by ~50% after tamoxifen injection at 8 weeks (Supplementary Fig. 6d, e). Without CRS treatment, Gls1-cKO mice did not display depression-like behaviors either before or after tamoxifen injection in two separate cohorts (Supplementary Fig. 7). More importantly, microglial Gls1 knockout abrogated CRS-induced depression-like behaviors, ascertained by the reduced immobility in TST (Fig. 1d), enhanced sucrose preference in SPT (Fig. 1e), and increased spending times, entry numbers, and travel distances in center zone in open field test (OFT) (Fig. 1f) without affecting motor ability of CRS-treated Gls1-cKO mice (Supplementary Fig. 8).

We next examined the roles of Gls1 in the regulation of microglial dysfunction in CRS mice. Immunohistochemical analyses demonstrated that microglial Gls1-cKO reversed CRS-induced increases in the number of Iba1^+^ cells in hippocampi (Fig. 1g, h) and prefrontal cortexes (Supplementary Fig. 9a, b). However, microglial Gls1-cKO showed no effects on inflammatory responses of microglia, ascertained by no significant difference regarding the protein levels of pro-inflammatory cytokines (TNF-α and IL-6) and the transcript levels of pro-inflammatory genes (*Tnf*, *Nos2*, and *Nfkb*) between CRS-induced Gls1-cKO and CRS control mice (Supplementary Fig. 10). We then determined the influence of Gls1 on microglia-mediated synapse damage. Immunohistochemical analyses revealed that the accumulation of CD68^+^ lysosomes and PSD95^+^ puncta within microglia in both dorsal hippocampal (Fig. 1i–l) and prefrontal cortical tissues (Supplementary Fig. 9c–f) of CRS mice were suppressed by microglial Gls1-cKO. Western blotting results also showed that microglial Gls1 deficiency reversed the CRS-induced reduction of PSD95 in the hippocampi (Fig. 1m, n) and prefrontal cortexes (Supplementary Fig. 9g, h). We also examined the concentration of corticosterone in the brains to determine the effects of Gls-cKO on potential hormonal stress responses induced by CRS. However, no significant difference in corticosterone concentration among groups was observed (Supplementary Fig. 11). Besides, no effect of Gls1-cKO was observed on blood cells (Supplementary Fig. 12). Together, our results showed the key roles of Gls1 in microglial activation and abnormal engulfment of synaptic structures under CRS conditions, implying Gls1 as an antidepressant target of MDD.

To test our premise, we treated mice with different doses (3, 10, 30 mg/kg) of Gls1 inhibitor CB839 via intraperitoneal injection (3 injections every other day) right after CRS exposure (Supplementary Fig. 13a). PBS injection was done on blank and CRS controls to avoid the influence of injection-induced stress. Behavior tests were performed two days after the third injection. CB839 injection reduced the immobility of CRS mice in TST (Fig. 1o), and increased the sucrose preference of CRS mice in SPT (Fig. 1p). CB839 injection also improved the performance of CRS mice in OFT, including increased spending times, entry numbers, and travel distance in center zone (Fig. 1q), without affecting motor ability (Supplementary Fig. 13b, c). Moreover, immunohistochemical analysis revealed that CB839 injection significantly reduced the number of Iba1^+^ cells, inhibited the accumulation of CD68^+^ lysosomes or PSD95^+^ puncta within microglia, and abrogated CRS-induced PSD95 expression declines in the dorsal hippocampi (Fig. 1r–y) and prefrontal cortexes (Supplementary Fig. 14) of CRS mice. CB839 injection also reversed the enhanced glutamate production in both the hippocampal and prefrontal cortical tissues of CRS mice (Supplementary Fig. 15). Hence, CB839 abrogated CRS-induced glutamine metabolism in the brains, implying that Gls1 regulates the synaptic pruning capacity of microglia through modulating the metabolic status of microglia.^4,8^ Besides, CB839 injection demonstrated negligible effects on the expression of pro-/anti-inflammatory genes (Supplementary Fig. 16) in the hippocampi of CRS mice. No effect of CB839 treatment was observed on blood cells (Supplementary Fig. 17). Hence, our results suggested that CB839 partially rescued CRS-induced depression-like behaviors and microglia-mediated synaptic damage, revealing Gls1 as a promising therapeutic target of MDD. Notably, since CB839 may affect various types of brain cells, we cannot exclude that CB839 exerts therapeutic effects not exclusively through microglial regulation, which requires further investigations in the future.

In summary, we demonstrated a significant increase in microglial Gls1 expression levels in CRS mouse brains. The microglial Gls1 knockout reversed depression-like behaviors presumably through inhibiting microglial activation and abnormal engulfment of synaptic structure (Supplementary Fig. 18). More importantly, the administration of Gls1 inhibitor CB839 abrogated CRS-induced depression-like behaviors and microglial dysfunction. Our study provides new evidence to support the link between microglial dysfunction and the pathogenesis of MDD and identifies microglial Gls1 as a promising target for the development of novel anti-depression therapy.

### Supplementary information

Supplemental materials

T & F-values for all figures

## Data Availability

All datasets generated for this study are included in the manuscript/Supplementary Files.

## References

[1] Yirmiya R, Rimmerman N, Reshef R (2015). Depression as a microglial disease. Trends Neurosci..

[2] Jia X, Gao Z, Hu H (2021). Microglia in depression: current perspectives. Sci. China Life Sci..

[3] Fu S (2022). ROS-targeted depression therapy via BSA-incubated ceria nanocluster. Nano Lett..

[4] Ding L (2021). Glutaminase in microglia: A novel regulator of neuroinflammation. Brain Behav. Immun..

[5] Gao G (2020). Glutaminase 1 regulates neuroinflammation after cerebral ischemia through enhancing microglial activation and pro-inflammatory exosome release. Front. Immunol..

[6] Gao G (2019). Glutaminase C regulates microglial activation and pro-inflammatory exosome release: relevance to the pathogenesis of Alzheimer’s disease. Front. Cell. Neurosci..

[7] Ji C (2022). Microglial glutaminase 1 deficiency mitigates neuroinflammation associated depression. Brain Behav. Immun..

[8] Palmieri EM (2017). Blockade of glutamine synthetase enhances inflammatory response in microglial cells. Antioxid. Redox. Signal..

[9] Seo JS, Mantas I, Svenningsson P, Greengard P (2021). Ependymal cells-CSF flow regulates stress-induced depression. Mol. Psychiatry.

