# Supplementary material for: Microglial glutaminase 1 mediates chronic restraint stress-induced depression-like behaviors and synaptic damages
Source: Signal Transduct Target Ther. 2023 Dec 15;8:452. doi: 10.1038/s41392-023-01699-8 (PMC10721840; doi:10.1038/s41392-023-01699-8)
Supplement: Supplementary file 1 — Supplemental materials [file 41392_2023_1699_MOESM1_ESM.docx]

Supplementary Materials for

Microglial glutaminase 1 mediates the chronic restraint stress-induced depression-like behaviors and neuroinflammation

Huili Chen^1ǂ^, Shengyang Fu^1ǂ^, Xiangyu Li^ǂ^, Meng Shi^ǂ^, Jiazhen Qian^1^, Shu Zhao^1^, Ping Yuan^2^, Lu Ding^1^, Xiaohuan Xia^1,3*^and Jialin C. Zheng^1,3*^

^1^Center for Translational Neurodegeneration and Regenerative Therapy, Tongji Hospital affiliated to Tongji University School of Medicine, Shanghai 200065, China. ^2^Department of Cardio-Pulmonary Circulation, Shanghai Pulmonary Hospital, School of Medicine, Tongji University, Shanghai, China. ^3^Translational Research Institute of Brain and Brain-Like Intelligence, Shanghai Fourth People's Hospital affiliated to Tongji University School of Medicine, Shanghai, 200434, China.

Correspondence to: [xiaohuan_xia1@163.com](mailto:xiaohuan_xia1@163.com); [jialinzheng@tongji.edu.cn](mailto:jialinzheng@tongji.edu.cn).

**This PDF file includes:**

Materials and Methods

Supplementary Figures. S1 to S18

**Methods and Materials**

**Animals**

Adult male (8–16 weeks of age) C57BL/6J mice and transgenic mice were used for establishing the chronic restraint stress (CRS) depression model. Four or five mice were housed per cage under a 12-h light-dark cycle (light on from 7 a.m. to 7 p.m.) and weighted every week with free access to food and water.

Gls1^flox/+^ transgenic mice were generated from Gls^tm1a(KOMP)Mbp^ “knockout first” germline-competent C57BL/6N embryonic stem cells that were purchased from the Knockout Mouse Project in which exon10 and exon11 of *Gls1* gene were flanked by loxP sites.^1^ Csf1r^CreER^ transgenic mice were purchased from the Jackson Laboratory (FVB-Tg(Csf1r-cre/Esr1*)1Jwp/J). Gls1^flox/+^ mice and Csf1r^CreER^ mice were crossed to generate Gls1 conditional knockout mouse line (Gls1-cKO). Gls1-cKO mice were validated by genotyping using the genomic DNA isolated from their tails. Control mice were Csf1r^CreER+^::Gls1^WT^. Mice were injected with tamoxifen (75 mg/kg, ip, 5 days, Sigma Aldrich, cat#T5648) to knockout Gls1 in microglia.

All mice studies and experimental procedures were approved by the Institutional Animal Care and Use Committee (IACUC) of Tongji University School of Medicine (reference number: SYXK (HU) 2014-0026).

**Chronic restraint stress (CRS)**

Mice were subjected to CRS by being gently placed in 50 ml conical tubes with eight holes for air flow for 4 h per day for 14 consecutive days. There is no food or water supply during the restraint phase. Mice received CRS were housed in separate cages. The tubes were cleared and dried every day after use. During the restraint phase, control mice were handled by hands of experimenter for 5 min and kept in their home cage without food or water supply.

**Behavioral assays**

All behavioral assays were performed on mice 10-16 weeks old. All behavior tests were performed during the light phase in a quiet room. All the behavioral analyses were performed by a researcher who were blinded to experimental conditions.

*Open field test (OFT)*.

OFT was performed under the bright interior lighting for researchers to evaluate the exploratory and locomotor behaviors of the mice. Briefly, mice were placed in the center of an arena (40 cm × 40 cm × 40cm) and allowed to explore freely in a quiet room for 5 min. The performances of the mice including the total movement distance, the time spent in center, and the number of crossing the center in OFT were recorded by a camera about 120 cm above the apparatus. 75% ethanol solution was used to clear the arena after each mouse finished the trial to avoid cross-influence between mice.

*Forced swim test (FST).* Mice were individually placed in a cylinder (20 cm diameter, 30 cm height) of water (23-25 °C) and forced to swim for 6 mins under normal light. Water depth was set to prevent mice from touching the bottom with their tails or hind limbs. Mice behaviors were recorded from the upward side. The immobile time and activity of each mouse during the last 4-min test was counted by smart real- time software.

*Sucrose preference test (SPT).* Mice were singly housed and habituated with two bottles of water for 1 day, followed by two bottles of 2% sucrose for 1 day. Mice were then water deprived for 24 h and then exposed to one bottle of 2% sucrose and one bottle of water for 2 h. Bottle positions were switched after 1 h (for a 2 h test). Total consumption of each fluid was measured and sucrose preference was defined as the average sucrose consumption ratio during the first and second hours. Sucrose consumption ratio was calculated by dividing the total consumption of sucrose by the total consumption of both water and sucrose.

*Tail suspension test (TST).* Mice were hanged upside down in the equipment for 6 min by tail with a camera at the side to record the behaviors of each mouse. The immobile time and activity of each mouse during the last 4-min test was counted and analyzed by a real- time smart analyzing software.

**Western blotting**

Animals were sacrificed right after behavior tests. Mice were anesthetized with 2% pentobarbital sodium (IP) and their brains were removed rapidly. The prefrontal cortex (PFC) and hippocampus were immediately dissected on ice and were homogenized in T-PER Protein Extraction Reagent (Thermo Scientific) containing a protease inhibitor cocktail (Sigma). The lysates were centrifuged at 15,000 g for 20 min at 4℃, then supernatants were mixed with 6× SDS loading buffer and boiled at 95℃ for 10 min. The protein concentration was determined via the BCA method using BCA kits (Thermo Scientific).

The prepared protein samples were separated by 10%-15% SDS-PAGE gel and transferred to a PVDF membrane. The membrane was blocked in 5% nonfat milk and subsequently incubated overnight with the primary antibodies including PSD95 (Cell signaling technology, cat # 3450s, rabbit, 1: 1000), synaptophysin 1 (Synaptic system, cat #101002, rabbit, 1: 1000), GLS1 (Proteintech, cat #66265-1-Ig, rabbit, 1:1000), TNF-α (Abcam, cat # Ab183218, rabbit, 1:1000), IL-6 (Abmart, cat # PY6089, rabbit, 1:1000) and β-actin (mouse, cat # a5441, Sigma, 1:5000) at 4℃. Relevant horseradish peroxidase (HRP)-conjugated secondary antibodies (1:5000) were incubated, and an electrochemiluminescence (ECL) detection reagent was applied to the PVDF membrane. The protein signal was detected using Pierce ECL Western Blotting Substrate (Thermo Fisher Scientific, Waltham, MA, United States).

**Quantitative real-time polymerase chain reaction (qRT-PCR).**

The fresh brain tissues were homogenized in QIAzol Lysis Reagent (QIAGEN). Total RNA was extracted using miRNeasy (QIAGEN) Mini Kits and quantified by Nanodrop. cDNA was obtained using a one-step first strand cDNA synthesis kit (HiScript III All-in-one RT SuperMix Perfect for qRT-PCR, Vazyme). The relative amount of target gene was calculated with qRT-PCR on a Step One platform (Applied Biosystems, Foster City, CA, USA). qRT-PCR was performed in a total reaction volume of 20 μl containing 10 μl SYBR Green Master mix (Taq Pro Universal SYBR qRT-PCR Master Mix, Vazyme) for 40 cycles (15 s at 95 °C and 1 min at 60 °C). Transcripts were amplified using specific primer sets for *Gapdh* (Forward: CATGTTCCAGTATGACTCCACTC, Reverse: GGCCTCACCCCATTTGATGT), *Nos2* (Forward: CCCTTCAATGGTTGGTACATGG, Reverse: ACATTGATCTCCGTGACAGCC), *Tnf* (Forward: ACGTGGAACTGGCAGAAGAG, Reverse: GGTCTGGGCCATAGAACTGA), *Nfkb* (Forward: CCTCTCTCGTCTTCCTCCAC, Reverse: GTTTGCGGAAGGATGTCTCC), *Cd206* (Forward: TCTTTGCCTTTCCCAGTCTCC, Reverse: TGACACCCAGCGGAATTTC), *Ym1* (Forward: TCACAGGTCTGGCAATTCTTCTG, Reverse: ACTCCCTTCTATTGGCCTGTCC), *Arg1* (Forward: TTTTAGGGTTACGGCCGGTG, Reverse: CCTCGAGGCTGTCCTTTTGA). The mRNA levels of *Gapdh* were used as internal controls.

**Magnetic bead sorting-based isolation of microglia, astrocytes, and neurons**

Mice were anesthetized with 2% pentobarbital sodium (IP). Brains tissues were collected from euthanized mice. Pre-cooling D-PBS was used to clean the tissue and chop the brain tissue, according to the manufacturer’s instruction (Miltenyi Biotec cell dissociation kit #130-107-677). Brain tissues were then incubated with enzyme mixture at 37 ℃ for 30 min to obtain dissociated cells. D-PBS was used to re-suspend cell precipitation and appropriate volume of debris removal solution was added to evenly mix. D-PBS (4ml) was then added to stop the reaction, and the mixture was centrifuged at 4 ℃ 3000 g for 10 min. The cell suspension was divided into 3 layers. The top two layers were removed. The bottom layer was mixed with D-PBS and centrifuged at 4℃ 1000 g for 10 min. The supernatant was removed and an appropriate volume of erythrocyte lysate was added. Cells were incubated at 4℃ for 10 minutes. 10 mL D-PBS was added to stop the reaction, and the mixture was centrifuged at 4℃ 300 g for 10 min to remove the supernatant.

Magnetic bead antibody for microglial membrane surface marker (CD11b, Miltenyi Biotec # 130-126-725) was added into cell suspension and incubated at 4℃ for 15 minutes under dark conditions. The cells were eluted into the centrifuge tube and centrifuged at 4℃ and 300 g for 5 min to obtain microglial precipitation. Magnetic bead antibody for astroglial membrane surface marker (Glast, Miltenyi Biotec # 130-095-826) was then added to obtain astrocyte precipitation following the above procedure. Neurons (Miltenyi Biotec # 130-126-602) were obtained by adding magnetic bead antibody that depleted all non-neuronal cells to acquire highly purified neurons from mouse brain tissue.

**Immunofluorescence staining.**

Mice were anesthetized with 2% pentobarbital sodium and transcardially perfused with cold PBS and 4% paraformaldehyde. The brains were removed, fixed overnight in 4% paraformaldehyde and then transferred to 30% sucrose solution and cut into 30-μm coronal sections. Each group samples were incubated in permeabilization and blocking buffer (3% BSA, 10% donkey serum, and 1% Triton X-100 in PBS), subsequently incubated overnight with the primary antibody GLS1 (Abcam, cat # Ab125212, rabbit, 1:500,), IBA1 (Abcam, cat # Ab125212, goat, 1:500), CD68(Abcam, cat # Ab125212, rabbit, 1:500), PSD95 (Cell signaling technology, cat #3450s, rabbit, 1: 500). Next, the brain slices were washed with PBS and secondary antibodies (1:500) or DAPI (1:1000) were incubated. Immunofluorescence was observed under a confocal microscopy (FV3000, Olympus).

**Enzyme-linked Immunosorbent Assay (ELISA)**

Hippocampal tissue lysates were collected from mouse brains, and the concentration of pro-inflammatory cytokine TNF-α (cat # 50349-MNAE, Sino Biological) was measured with commercially available ELISA kits according to manufacturer’s protocols.

**Glutamate concentration analysis**

Glutamic acid test kit (Invitrogen, cat # A12221) was used for glutamic acid concentration test and the steps were followed according to the manufacture’s instruction. The standard product was diluted with gradients from 0 μM to 20 μM and added into the 96-well plate with 50 μl per well. Hydrogen peroxide (10 μM, 50 μl) was used as positive control. The reaction solution was added into the wells for standard substance, samples, and the positive control. The reaction solution was incubated at 37℃ for 30 min away from light. Excitation light and emission light with wavelength at 585 and 571 nm, respectively, were used to determine the reaction signals in the microplate reader. Glutamate concentration was calculated according to the curve obtained from the standard product.

**Statistical Analysis.** Data are expressed as the mean ± s.d. from at least two independent experiments. Statistical differences between the test and control values were analyzed using Student’s *t*-test. For multiple comparisons, statistical differences were analyzed by applying ordinary one-way ANOVA (Tukey’s multiple comparison test). Differences in data were considered statistically significant as follows: **p*<0.05, ***p*<0.01, ****p*<0.001, and *****p*<0.0001. Statistical analysis was performed using GraphPad Prism (version 9, GraphPad, San Diego, USA).

**
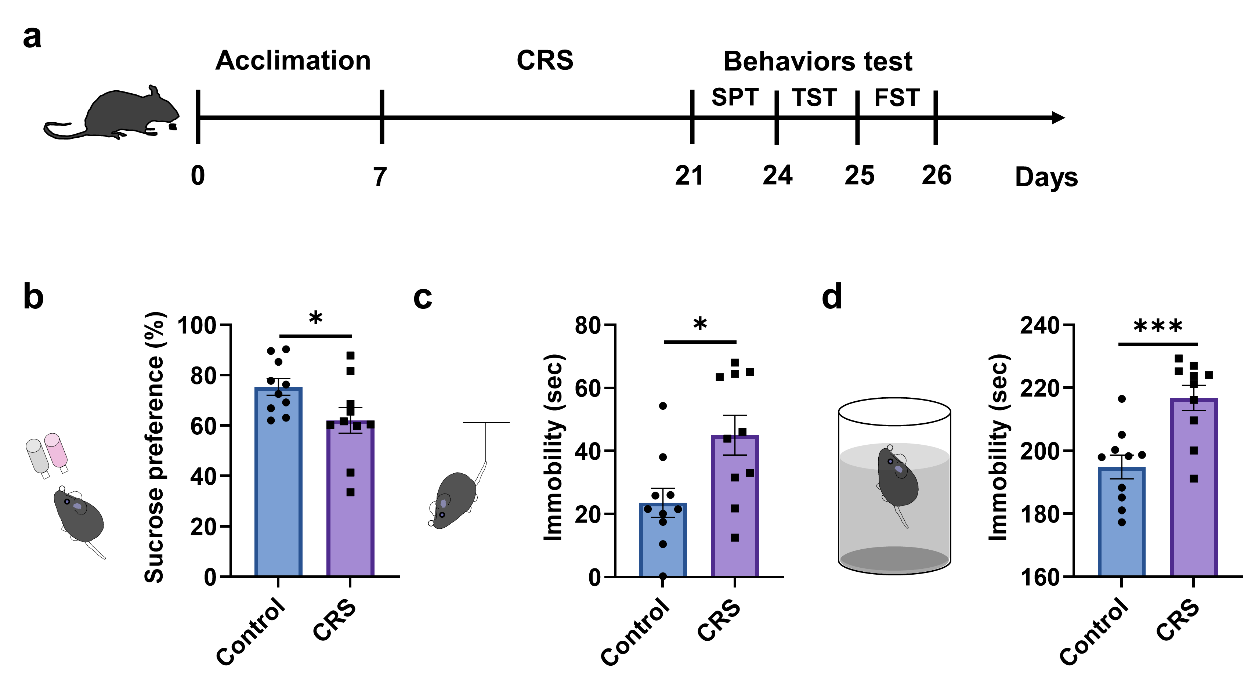
**

**Supplementary Fig 1. Establishment of chronic restraint stress model.**

(**a**) Experimental paradigms of depression-related behavior tests. (**b**) Performance of control or CRS-treated mice in sucrose preference test (n=10 animals). (**c**) Performance of control or CRS-treated mice in tail suspension test (TST) (n=10 animals). (**d**) Performance of control or CRS-treated mice in forced swimming test (FST) (n=10 animals). Error bars denote s.d. * and *** denote *p* < 0.05 and *p* < 0.001, respectively. The statistical difference among two groups was assessed with the unpaired two-tailed Student’s *t*-test.


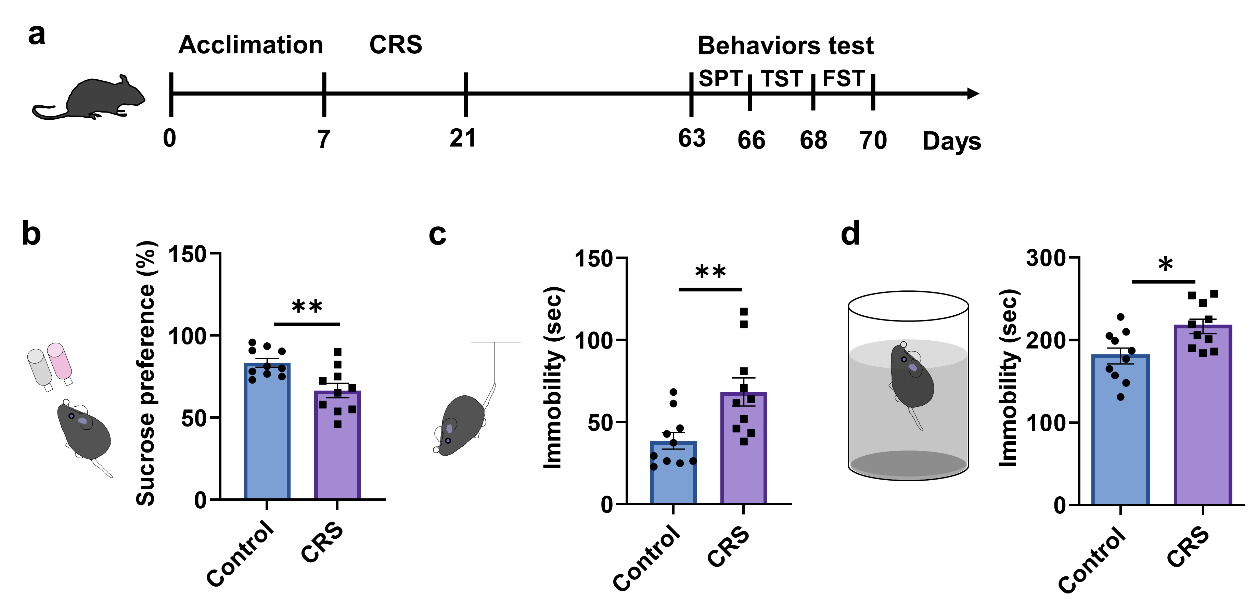


**Supplementary Fig 2. Endurance of depression-like behaviors 6 weeks post CRS.**

(**a**) Experimental paradigms of depression-related behaviortests 6 weeks post CRS. (**b**) Performance of ctrl or CRS-treated mice in SPT 6 weeks post CRS (n=10 animals). (**c**) Performance of ctrl or CRS-treated mice in TST 6 weeks post CRS (n=10 animals). (**d**) Performance of ctrl or CRS-treated mice in FST 6 weeks post CRS (n=10 animals). Error bars denote s.d. ** denotes *p* < 0.01. The statistical difference among two groups was assessed with the unpaired two-tailed Student’s *t*-test.


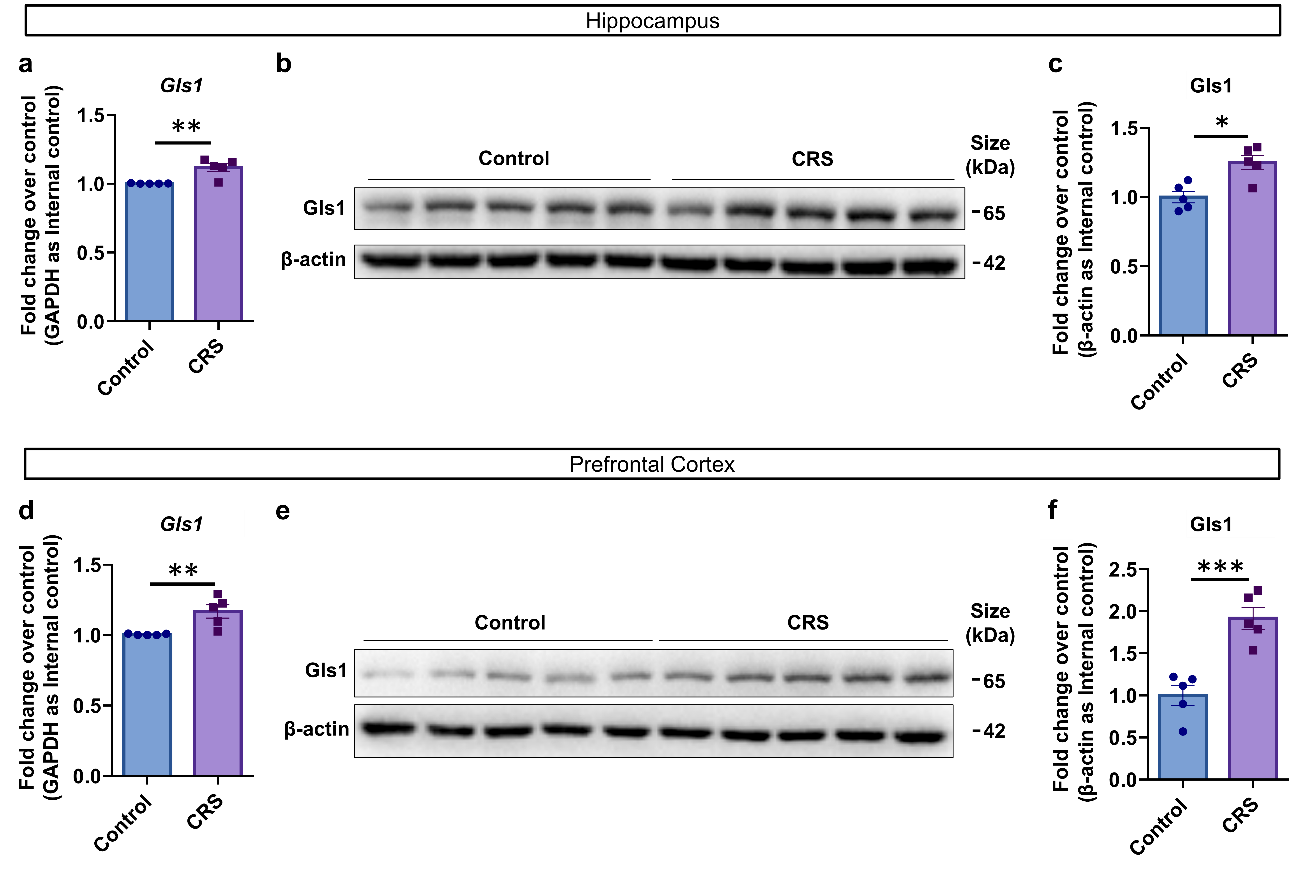


**Supplementary Fig 3. Up-regulation of Gls1 expression in CRS mouse brains.**

(**a**) qRT-PCR analyses of *Gls1* transcript expression levels in the hippocampus (n=5 animals). (**b**) Representative blot of GLS1 protein expression levels in the hippocampus (n=5 animals). (**c**) Quantification of GLS1 protein expression levels in the hippocampus (n=5 animals). (**d**) qRT-PCR analyses of *Gls1* transcript expression levels in the prefrontal cortex (n=5 animals). (**e**) Representative blot of Gls1 protein expression levels in the prefrontal cortex (n=5 animals). (**f**) Quantification of Gls1 protein expression levels in the prefrontal cortex (n=5 animals). Error bars denote s.d.. *, **, and *** denote *p* < 0.05, *p* < 0.01, and *p* < 0.001, respectively. The statistical difference among two groups was assessed with the unpaired two-tailed Student’s *t*-test.

**
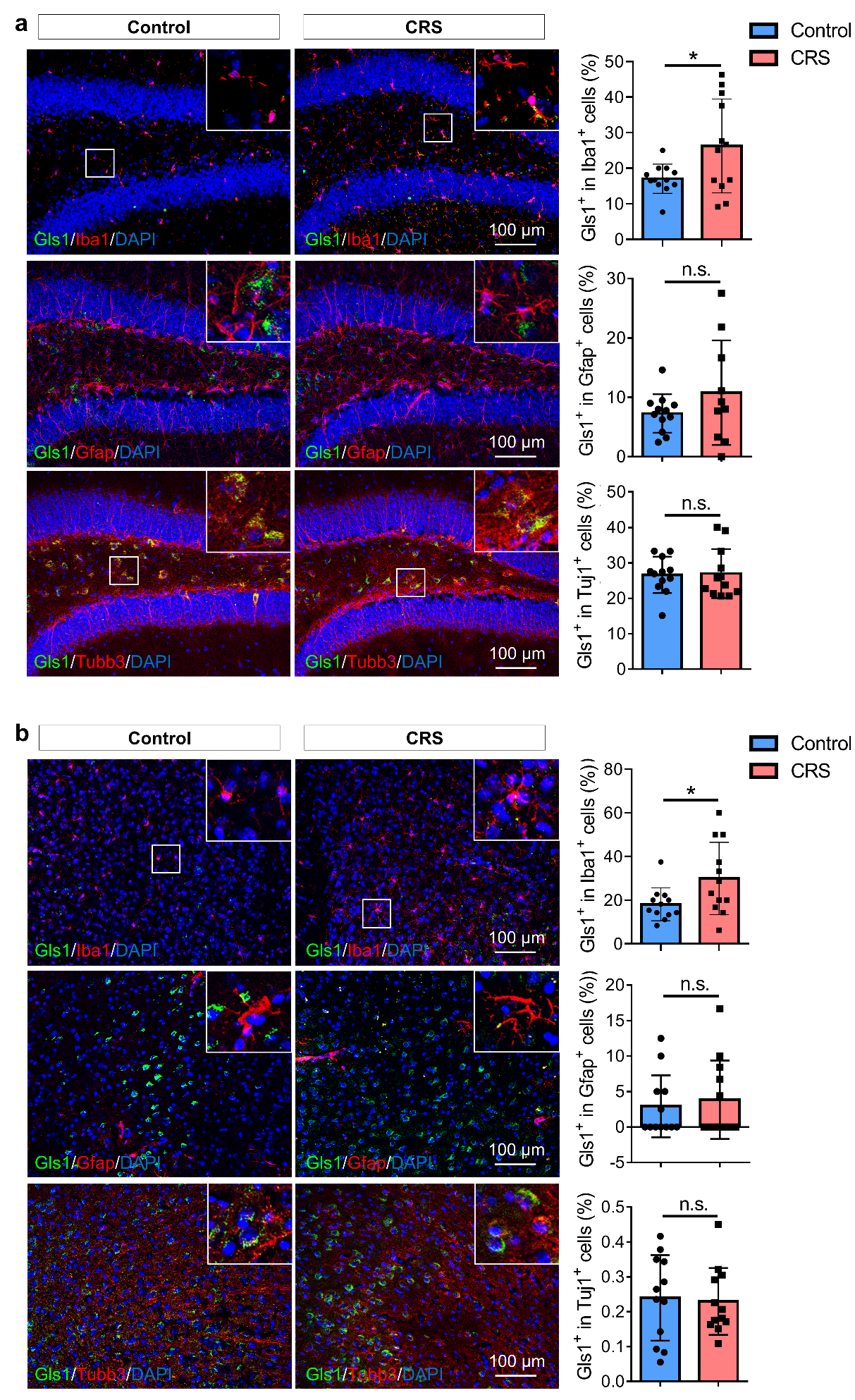
**

**Supplementary Fig 4. Excessive expression of Gls1 in microglia in the CRS mouse hippocampus.**

(**a**) Representative images of Gls1 immunostaining (green) and immunostaining of cell-specific markers Iba1/Gfap/Tuj1 (red) in the hippocampus of control and CRS mice. The quantification data of Gls1^+^ cells in Iba1^+^/Gfap^+^/Tuj1^+^ cells were provided in the right panels (n=12 slides, 2 slides/animal). (**b**) Representative images of Gls1 immunostaining (green) and immunostaining of cell-specific markers Iba1/Gfap/Tuj1 (red) in the prefrontal cortical tissues of control and CRS mice. The quantification data of Gls1^+^ cells in Iba1^+^/Gfap^+^/Tuj1^+^ cells were provided in the right panels (n=12 slides, 2 slides/animal). Error bars denote s.d. **** denotes p < 0.0001. The statistical difference among two groups was assessed with the unpaired two-tailed Student’s t-test.

**
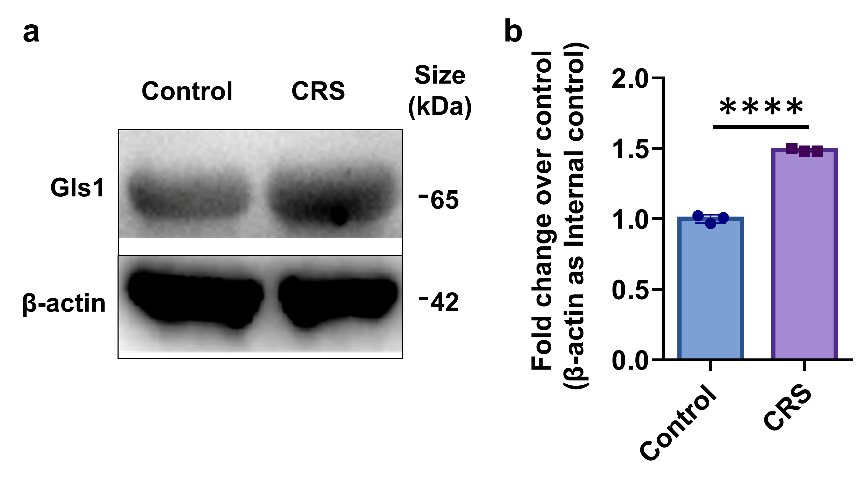
**

**Supplementary Fig 5. Endurance of microglial Gls1 excessive expression 6 weeks post CRS.**

(**a**) Representative blot of western blotting for the expression levels of Gls1 protein in CD11b^+^ microglia 6 weeks post CRS treatment. (**b**) Quantification of western blotting results (n=3, each dot indicates sorted cells from 10 animals). Error bars denote s.d. **** denotes *p* < 0.0001. The statistical difference among two groups was assessed with the unpaired two-tailed Student’s *t*-test.


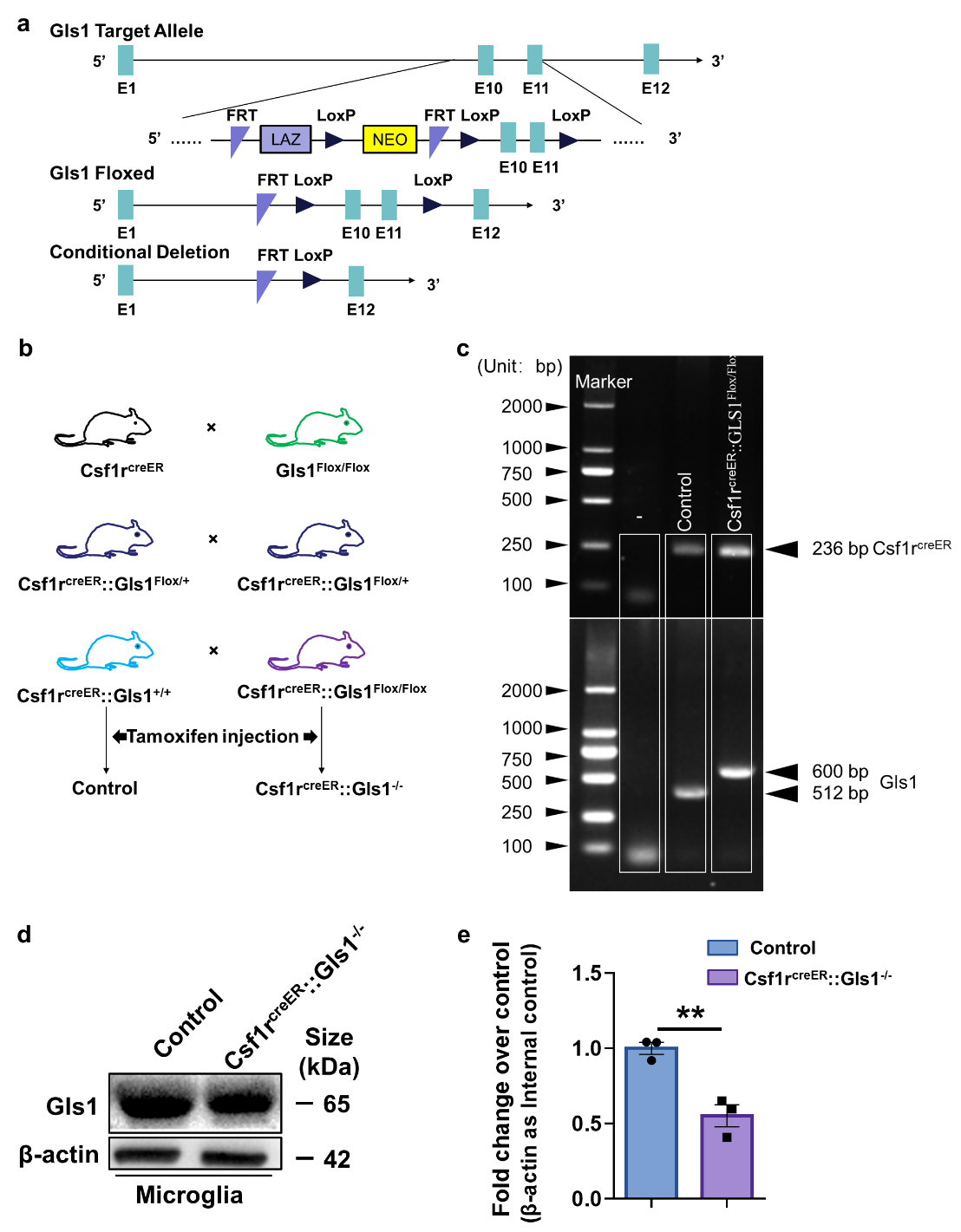


**Supplementary Fig 6.** **Generating microglial Gls1-Conditional knockout mice.**

(**a**) The experimental design for conditional knockout of Gls1 gene. (**b**) The procedures for mice crossing to get Gls1-cKO and control mice. (**c**) PCR images showing the genotyping of Gls1-cKO and control mice. (**d**) Microglia were isolated from control and Gls1-cKO mice (after tamoxifen injection) by Magnetic beads (marked by CD11b) and microglial Gls1 expression were detected by western blotting. (**e**) Microglial Gls1 was down-expressed in Gls1-cKO mice after tamoxifen injection (n=3, each dot indicates sorted cells from 10 animals). All data are represented as means ± SEM. ** denotes *p* < 0.01. The statistical difference among two groups was assessed with unpaired two-tailed Student’s *t*-test.


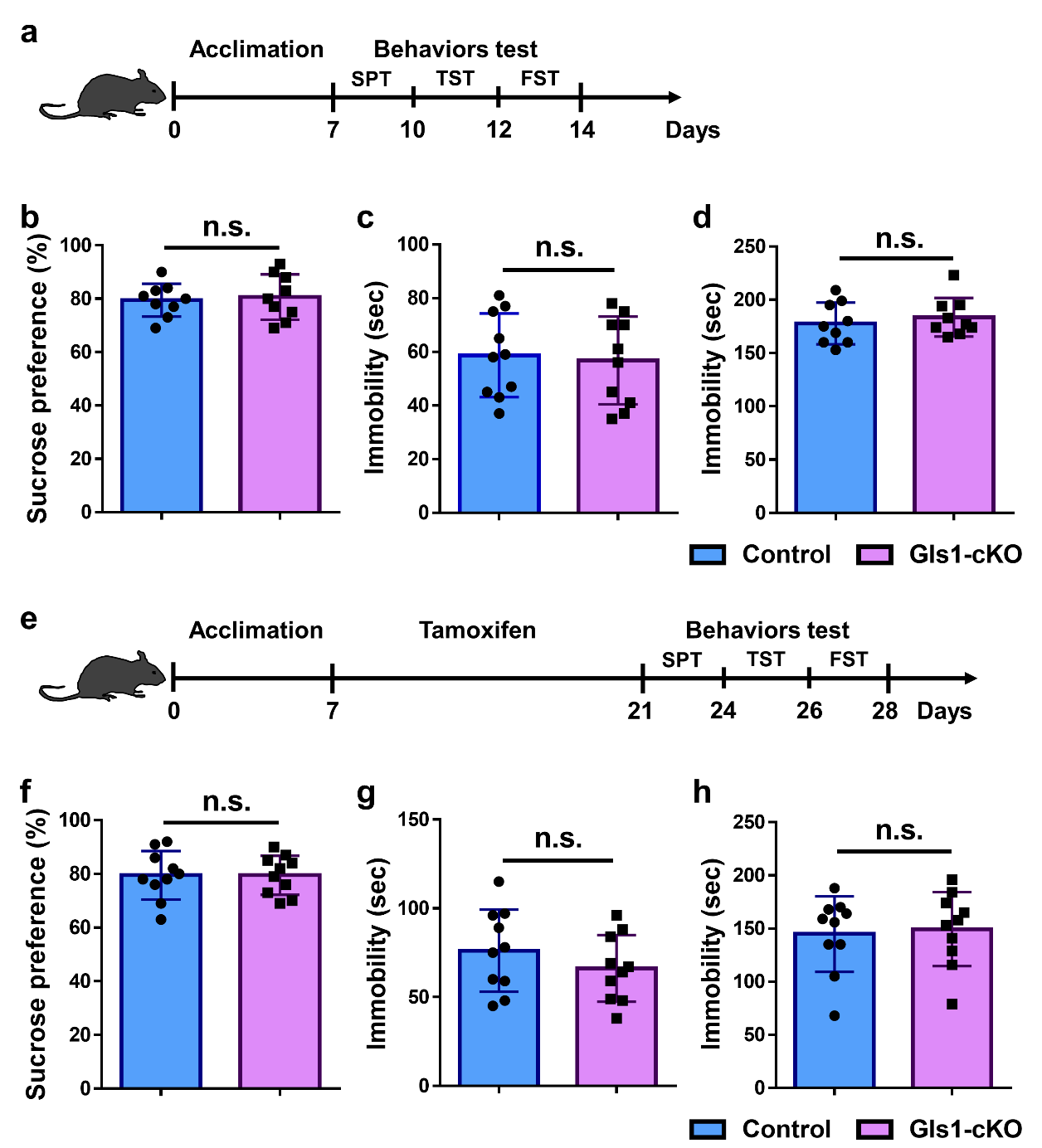


**Supplementary Fig 7. Depression-related performance of microglial Gls1-cKO mice after tamoxifen injection.**

(**a**) The experimental schedule of transgenic mice study (before tamoxifen). (**b**) Performance of control and microglial Gls1-cKO mice in SPT before tamoxifen injection (n=10 animals, two-way ANOVA). (**c**) Performance of control and microglial Gls1-cKO mice in TST before tamoxifen injection (n=10 animals, two-way ANOVA). (**d**) Performance of control and microglial Gls1-cKO mice in FST before tamoxifen injection (n=10 animals, two-way ANOVA). (**e**) The experimental schedule of transgenic mice study (after tamoxifen). (**f**) Performance of control and microglial Gls1-cKO mice in SPT after tamoxifen injection (n=10 animals, two-way ANOVA). (**g**) Performance of control and microglial Gls1-cKO mice in TST after tamoxifen injection (n=10 animals, two-way ANOVA). (**h**) Performance of control and microglial Gls1-cKO mice in FST after tamoxifen injection (n=10 animals, two-way ANOVA). All data are represented as means ± s.d.. ns, non-statistical differences.

**
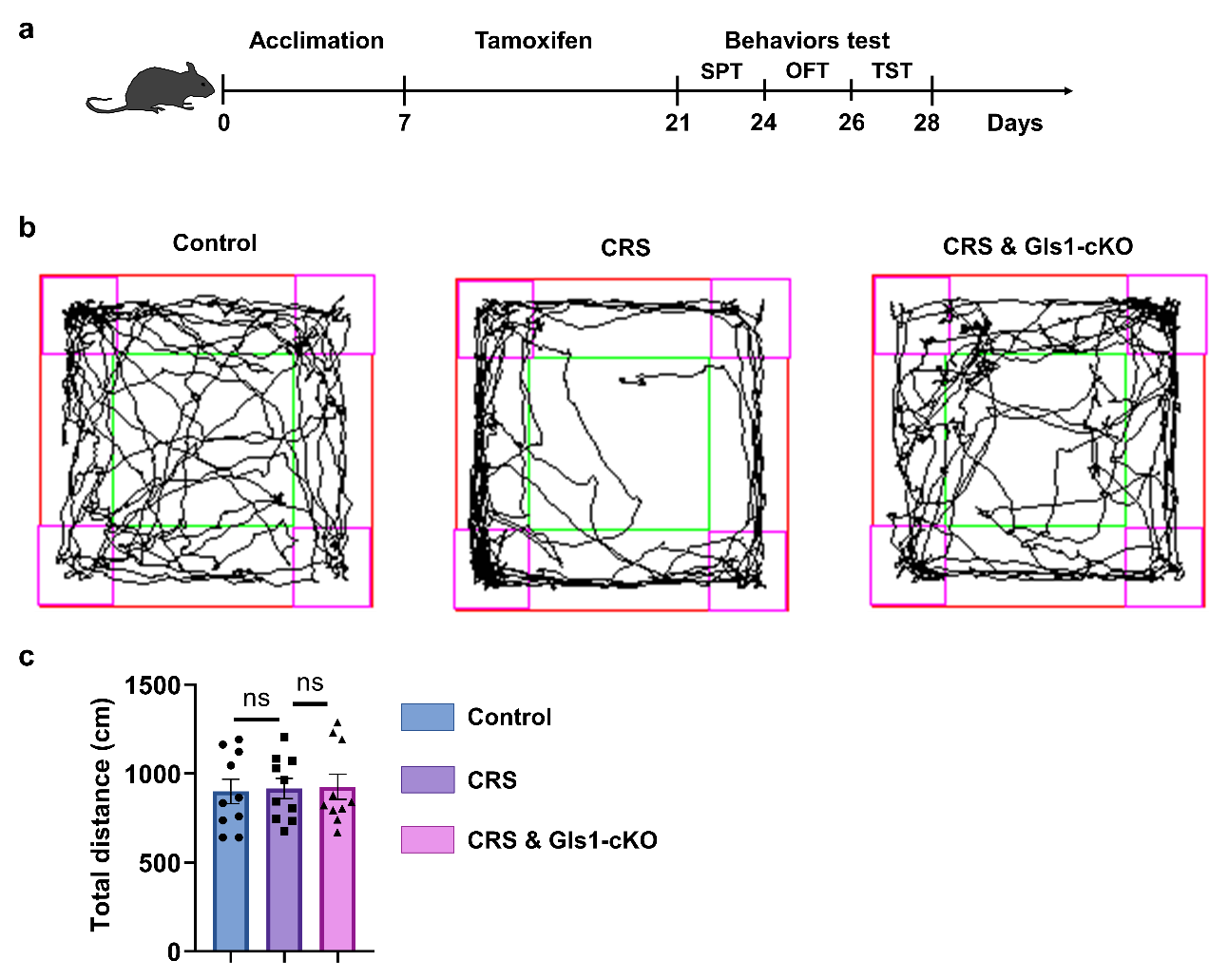
**

**Supplementary Fig 8. Depression-related performance of microglial Gls1-cKO mice in OFT.**

(**a**) The experimental schedule of transgenic mice study. (**b**) The moving track of control, CRS, and microglial Gls1-cKO CRS mice in the open field. (**c**) The total moving distance of control, CRS, and microglial Gls1-cKO CRS mice in the open field (n=10 animals). All data are represented as means ± s.d.. ns, non-statistical differences. The statistical difference among groups was assessed with the parametric one-way ANOVA with post-hoc Bonferroni test.

**
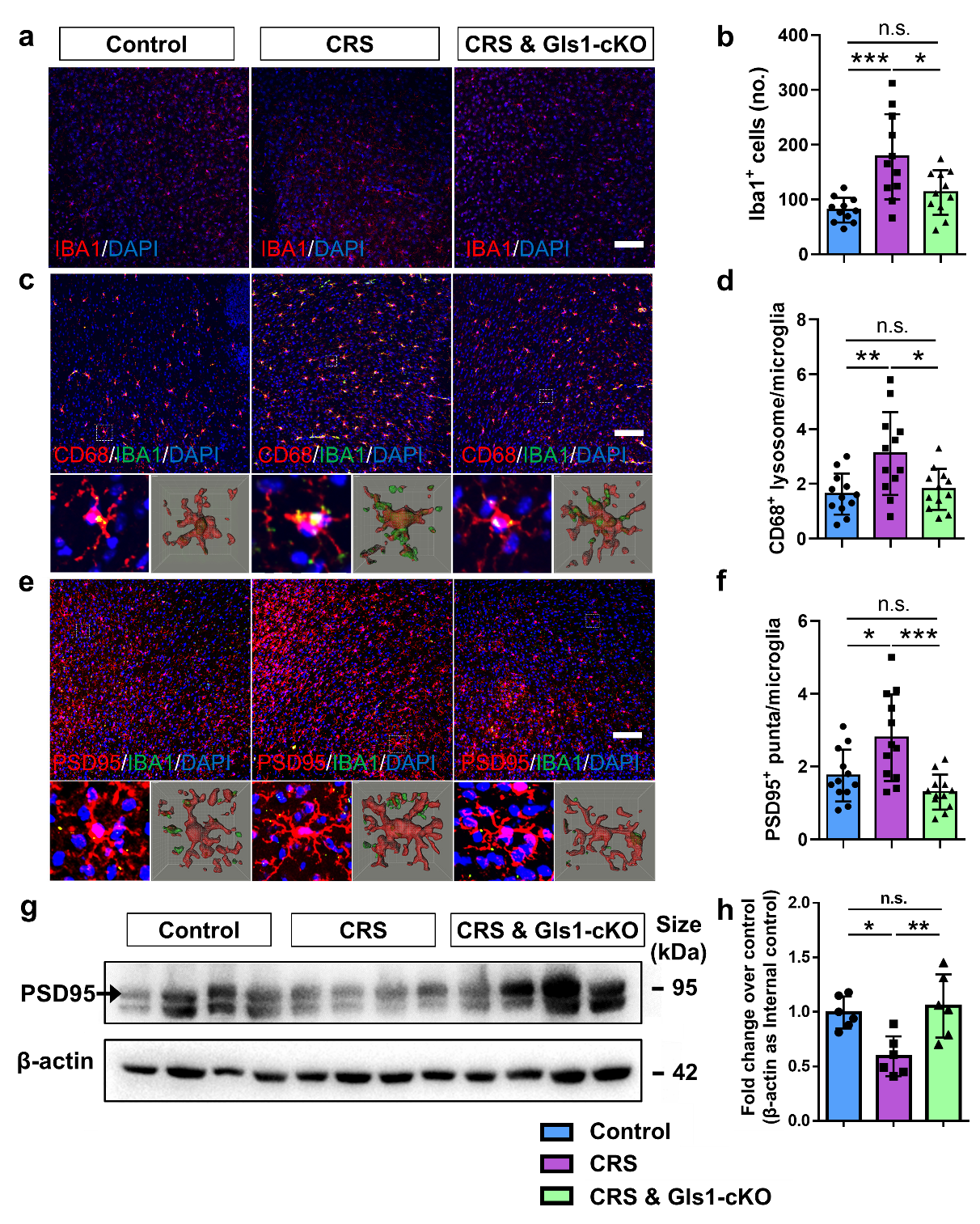
**

**Supplementary Fig 9. Microglial Gls1-cKO reverses CRS-induced microglial activation and excessive synaptic pruning.**

(**a**) Representative images of Iba1 immunostaining (green) of microglia in the prefrontal cortexes of control, CRS, and Gls1-cKO CRS mice. (**b**) Quantification of Iba1^+^ cell numbers (n=12 slides, 2 slides/animal, one-way ANOVA). (**c**) Representative images of Iba1 immunostaining (green) and CD68 immunostaining (red) of microglia in the prefrontal cortexes of control, CRS, and Gls1-cKO CRS mice. (**d**) Quantification of CD68^+^ lysosome numbers in Iba1^+^ cells n=12 slides, 2 slides/animal, one-way ANOVA). (**e**) Representative images of Iba1 immunostaining (green) and Psd95 immunostaining (red) of microglia in the prefrontal cortexes of control, CRS, and Gls1-cKO CRS mice. (**f**) Quantification of Psd95^+^ puncta numbers in Iba1^+^ cells (n=12 slides, 2 slides/animal, one-way ANOVA). (**g**) Representative blots for Psd95 protein expression in the prefrontal cortexes of control, CRS, and Gls1-cKO CRS mice. (**h**) Quantification results of western blotting analysis (n=6 animals, one-way ANOVA). Scale bar: 100 μm. The statistical difference among groups was assessed with the parametric one-way ANOVA with post-hoc Bonferroni test. All data are represented as means ± s.d.. n.s., not significant difference.


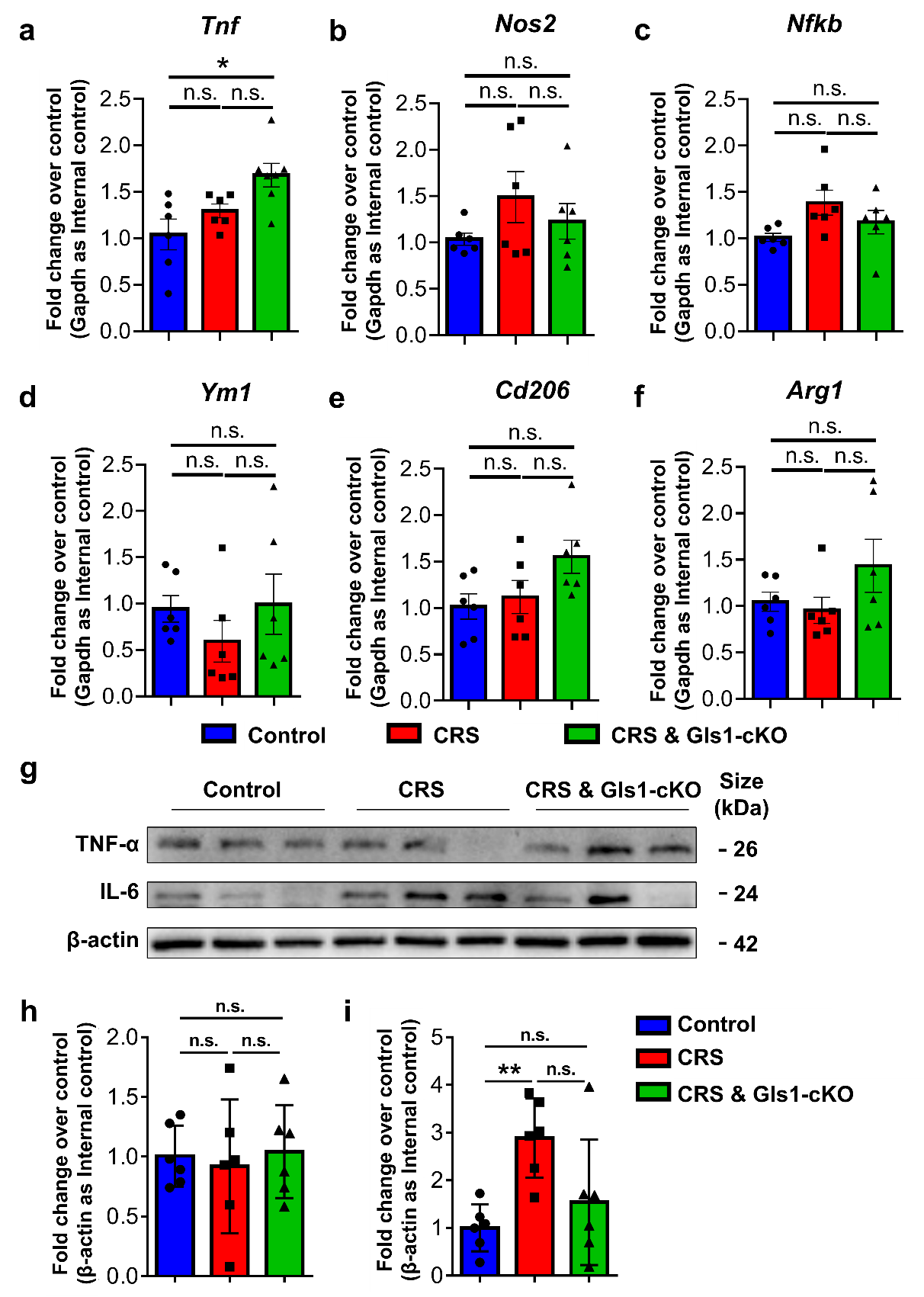


**Supplementary Fig 10. Microglial Gls1-cKO has no effects on inflammatory responses of CRS mice.**

(**a-c**) qRT-PCR analysis of pro-inflammatory genes *Tnf* (**a**), *Nos2* (**b**), and *Nfkb* (**c**) in the hippocampus of control, CRS, and microglial Gls1-cKO CRS mice (n=6 animals). (**d-f**) qRT-PCR analysis of anti-inflammatory genes *Ym1* (**d**), *Cd206* (**e**), and *Arg1* (**f**) in the hippocampus of control, CRS, and microglial Gls1-cKO CRS mice (n=6 animals). (**g**) Representative blot of western blotting for the expression levels of TNF-α and IL-6 proteins in the hippocampus of control, CRS, and microglial Gls1-cKO CRS mice. (**h, i**) Quantification of western blotting results for TNF-α (**h**) and IL-6 (**i**) (n=6 animals). The statistical difference among groups was assessed with the parametric one-way ANOVA with post-hoc Bonferroni test. All data are represented as means ± s.d.. n.s., not significant difference.


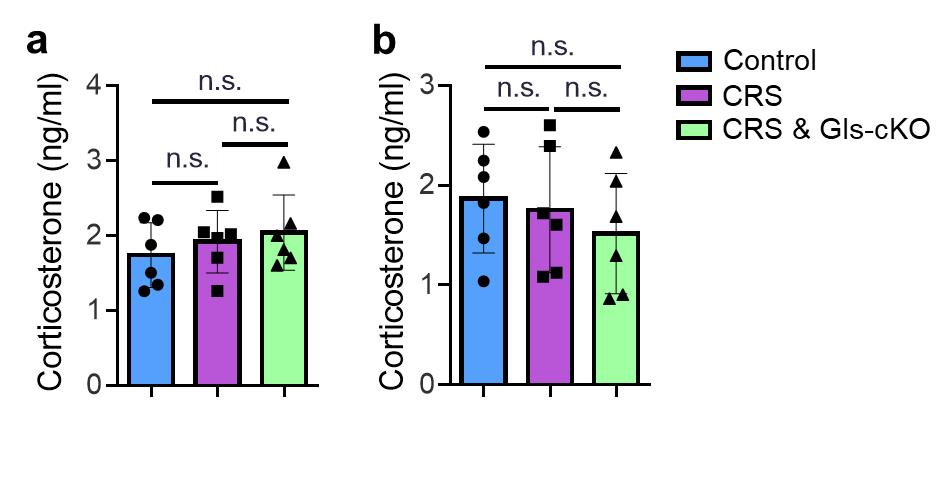


**Supplementary Fig 11. Microglial Gls1-cKO has no effects on corticosterone production of CRS mice.**

(**a**) Concentration of corticosterone in the hippocampi of control, CRS, and microglial Gls1-cKO CRS mice (n=6 animals, unpaired *t* test). (**b**) Concentration of corticosterone in the prefrontal cortical tissues of control, CRS, and microglial Gls1-cKO CRS mice (n=6 animals, one-way ANOVA). All data are represented as means ± s.d.. n.s., not significant difference.

**
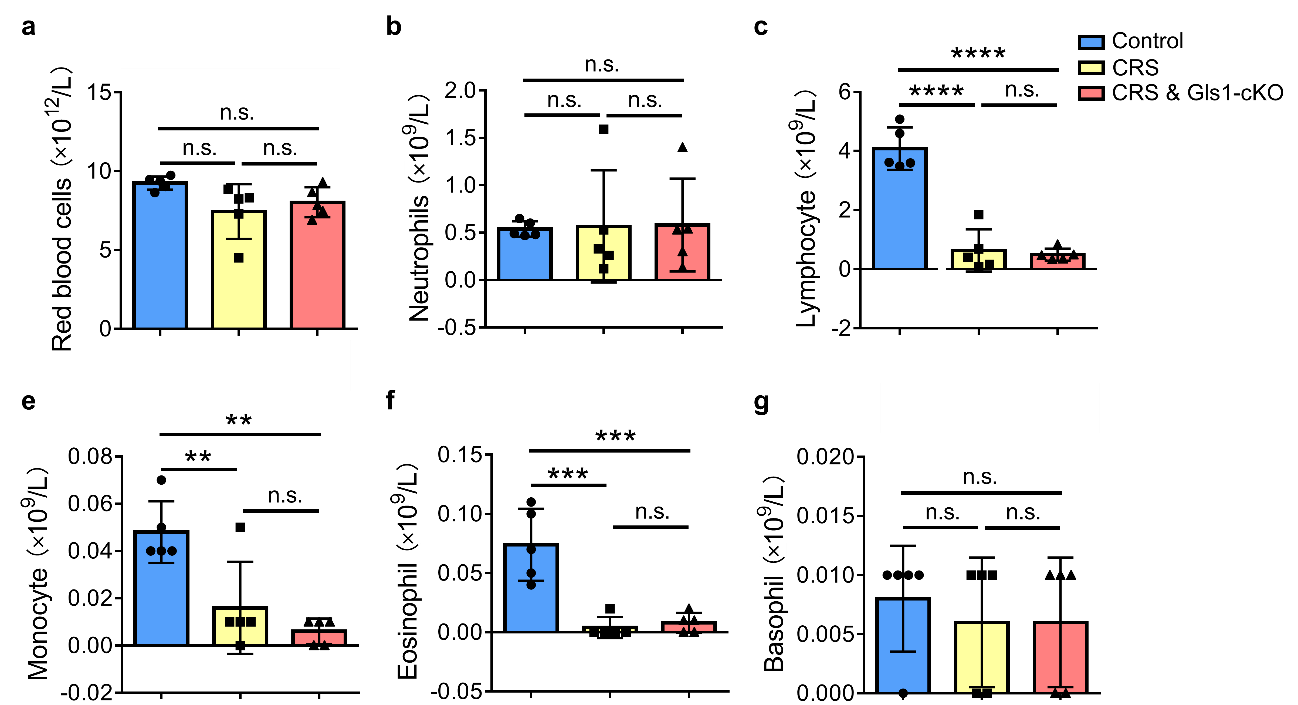
Supplementary Fig 12. Microglial Gls1-cKO has no effects on peripheral cells.**

(**a**) Number of red blood cells in blood (n=5 animals, one-way ANOVA). (**b**) Number of neutrophils in blood (n=5 animals, one-way ANOVA). (**c**) Number of lymphocytes in blood (n=5 animals, one-way ANOVA). (**d**) Number of monocytes in blood (n=5 animals, one-way ANOVA). (**e**) Number of eosinophils in blood (n=5 animals, one-way ANOVA). (**f**) Number of basophils in blood (n=5 animals, one-way ANOVA). All data are represented as means ± s.d.. n.s., not significant difference.


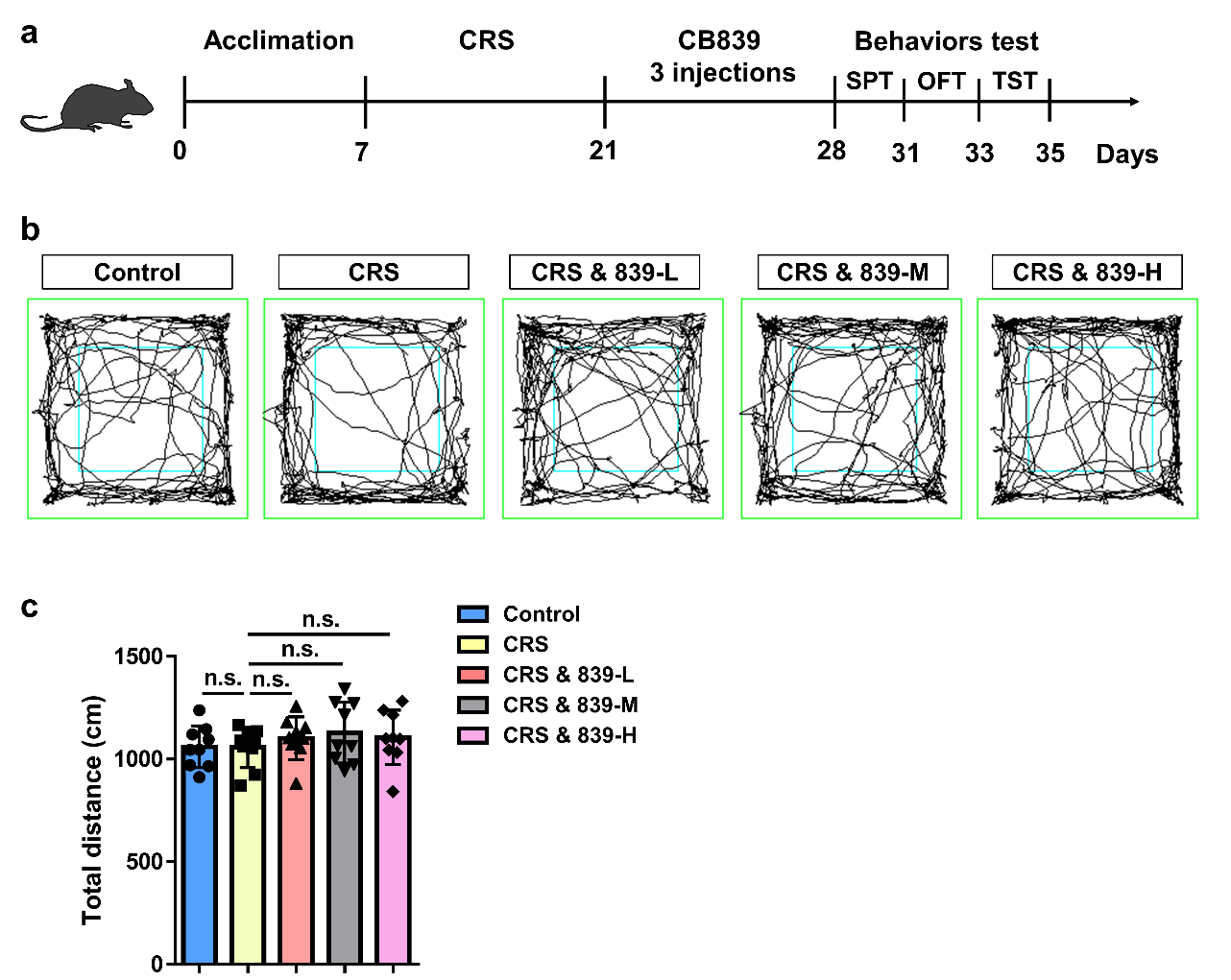


**Supplementary Fig 13. Depression-related performance of CB839 injected CRS mice in OFT.**

(**a**) The experimental schedule of CB839-application study. (**b**) The moving track of control, CRS, and different doses (L: 3 mg/kg, M: 10 mg/kg, and H: 30 mg/kg) of CB839-injected CRS mice in the open field. (**c**) The total moving distance of control, CRS, and different doses of CB839-injected CRS mice in the open field (n=9 animals, one-way ANOVA). All data are represented as means ± s.d.. ns, not significant difference.


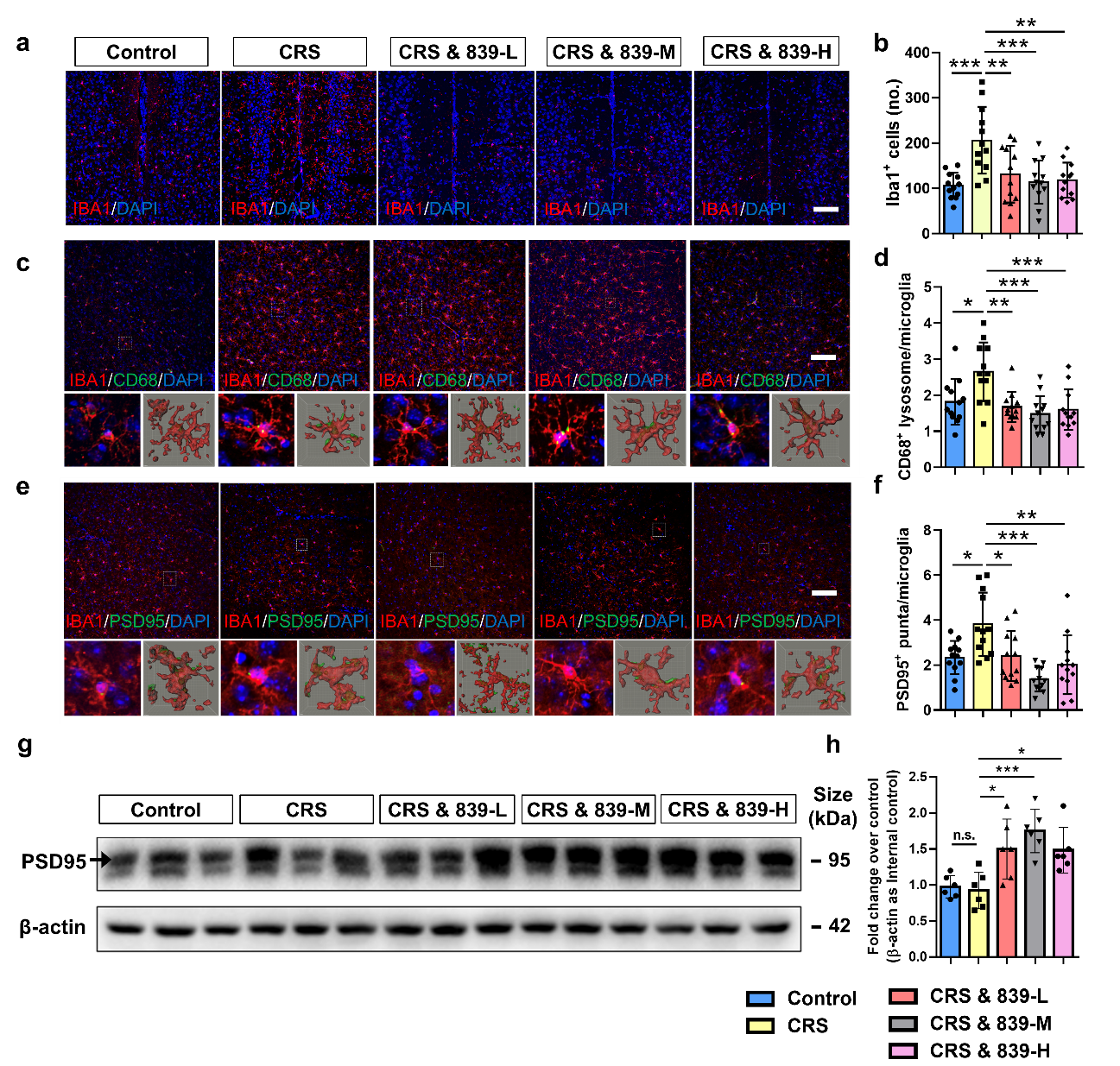


**Supplementary Fig 14. CB839 reverses CRS-induced microglial activation and excessive synaptic pruning.**

(**a**) Representative images of Iba1 immunostaining (red) of microglia in the prefrontal cortexes of control, CRS, and different doses (L: 3 mg/kg, M: 10 mg/kg, and H: 30 mg/kg) of CB839-injected CRS mice. (**b**) Quantification of Iba1^+^ cell numbers (n=12 slides, 2 slides/animal, one-way ANOVA). (**c**) Representative images of Iba1 immunostaining (green) and CD68 immunostaining (red) of microglia in the prefrontal cortexes of control, CRS, and different doses (L: 3 mg/kg, M: 10 mg/kg, and H: 30 mg/kg) of CB839-injected CRS mice. (**d**) Quantification of CD68^+^ lysosome numbers in Iba1^+^ cells n=12 slides, 2 slides/animal, one-way ANOVA). (**e**) Representative images of Iba1 immunostaining (green) and Psd95 immunostaining (red) of microglia in the prefrontal cortexes of control, CRS, and different doses (L: 3 mg/kg, M: 10 mg/kg, and H: 30 mg/kg) of CB839-injected CRS mice. (**f**) Quantification of Psd95^+^ puncta numbers in Iba1^+^ cells (n=12 slides, 2 slides/animal, one-way ANOVA). (**g**) Representative blots for Psd95 protein expression in the prefrontal cortexes of control, CRS, and different doses (L: 3 mg/kg, M: 10 mg/kg, and H: 30 mg/kg) of CB839-injected CRS mice. (**h**) Quantification results of western blotting analysis (n=6 animals, one-way ANOVA). Scale bar: 100 μm. The statistical difference among groups was assessed with the parametric one-way ANOVA with post-hoc Bonferroni test. All data are represented as means ± s.d.. n.s., not significant difference.


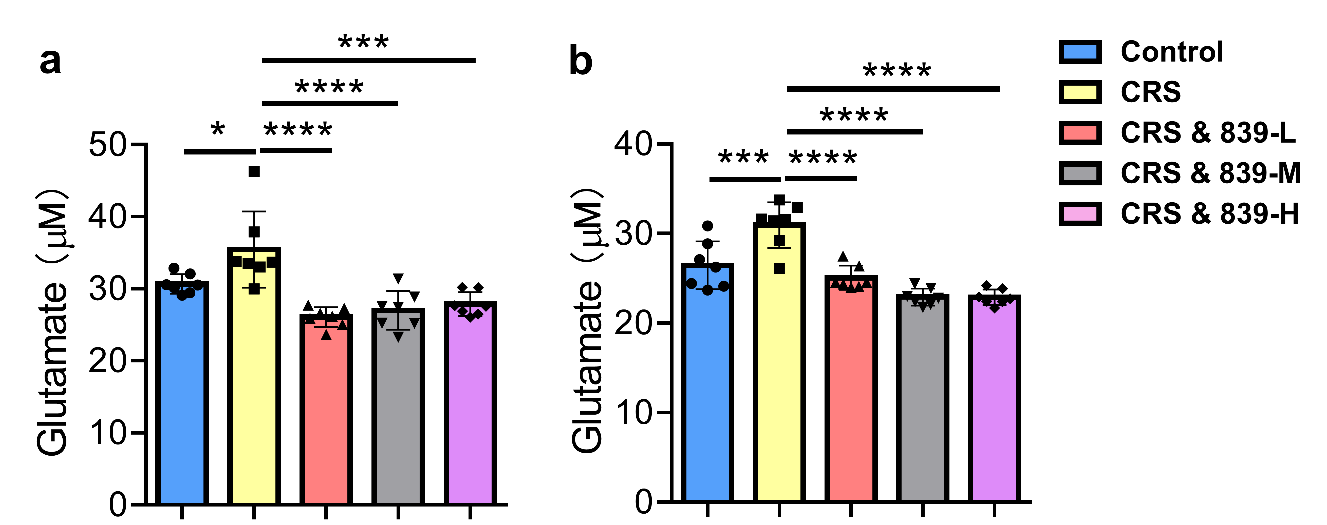


**Supplementary Fig 15. CB839 reveres CRS-induced glutamate hyper-production.**

(**a**) Concentration of glutamate in the hippocampi of control, CRS, and different doses (L: 3 mg/kg, M: 10 mg/kg, and H: 30 mg/kg) of CB839-injected CRS mice (n=7 animals). (**b**) Concentration of glutamate in the prefrontal cortical tissues of control, CRS, and different doses (L: 3 mg/kg, M: 10 mg/kg, and H: 30 mg/kg) of CB839-injected CRS mice (n=7 animals). The statistical difference among groups was assessed with the parametric one-way ANOVA with post-hoc Bonferroni test. All data are represented as means ± s.d..

**
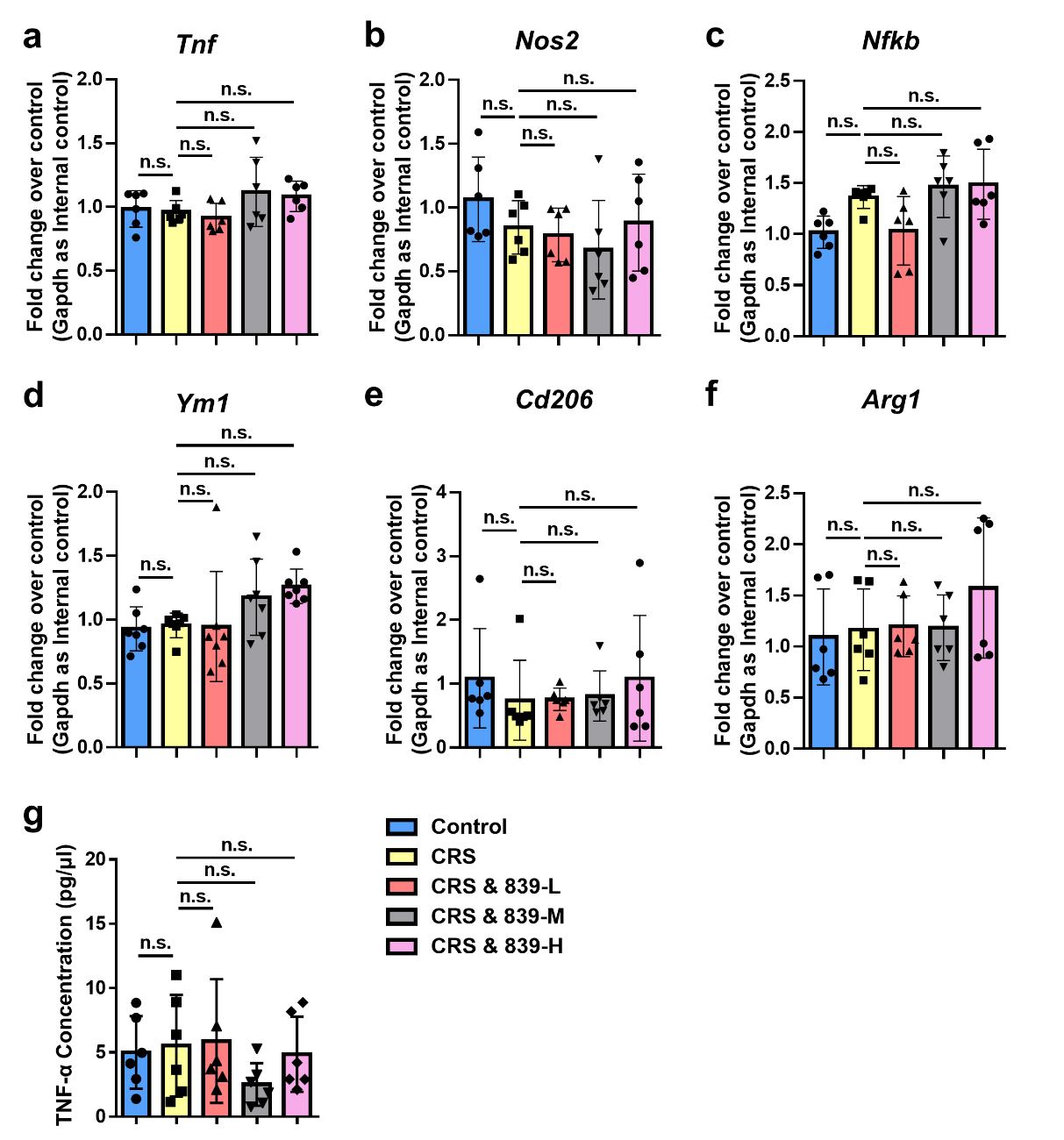
**

**Supplementary Fig 16. CB839 has no effects on inflammatory responses of CRS mice.**

(**a-c**) qRT-PCR analysis of pro-inflammatory genes *Tnf* (**a**), *Nos2* (**b**), *Nfkb* (**c**) in the hippocampus of control, CRS, and different doses (L: 3 mg/kg, M: 10 mg/kg, and H: 30 mg/kg) of CB839-injected CRS mice (n=8 animals). (**d-f**) qRT-PCR analysis of anti-inflammatory ones *Ym1* (**d**), *Cd206* (**e**), *Arg1* (**f**) in the hippocampus of control, CRS, and different doses (L: 3 mg/kg, M: 10 mg/kg, and H: 30 mg/kg) of CB839-injected CRS mice (n=8 animals). (**g**) ELISA analysis for the concentrations of TNF-α in the hippocampal lysates of control, CRS, and different doses of CB839-injected CRS mice (n=6 animals). The statistical difference among groups was assessed with the parametric one-way ANOVA with post-hoc Bonferroni test. All data are represented as means ± s.d.. n.s., not significant difference.


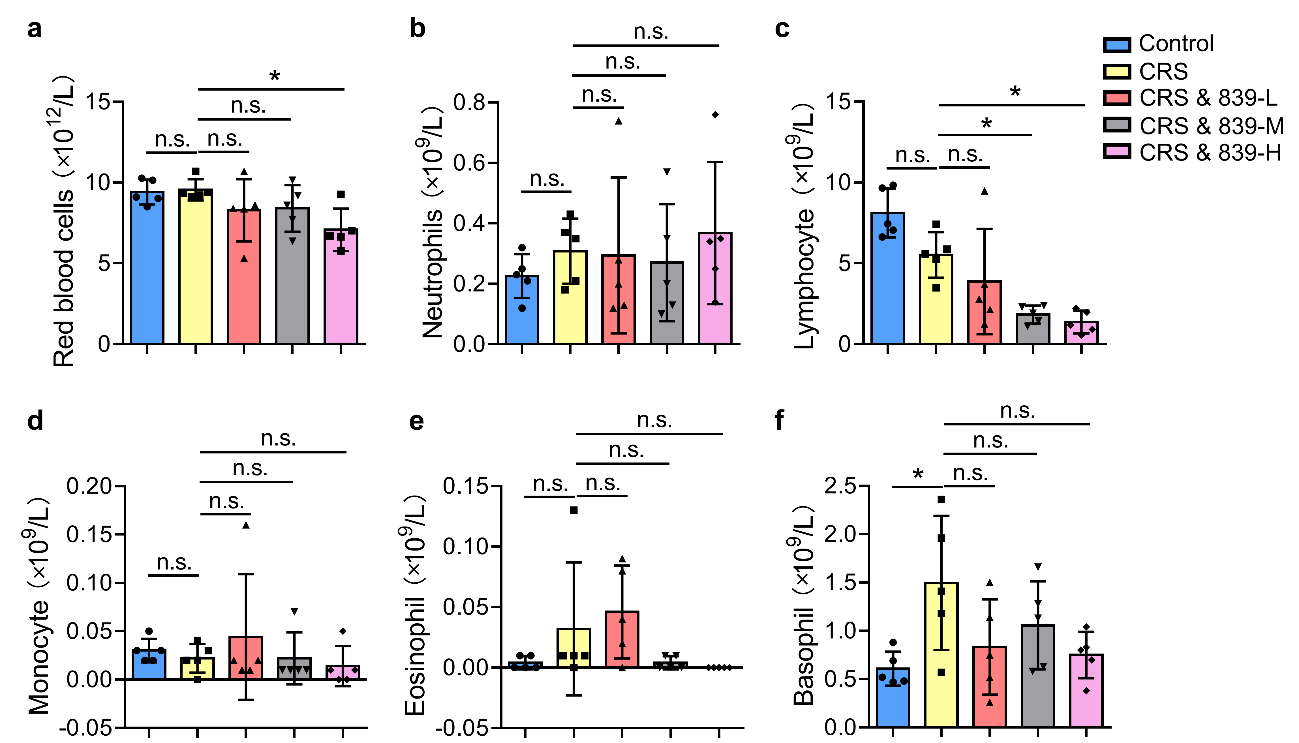


**Supplementary Fig 17. CB839 has no effects on most types of peripheral cells.**

(**a**) Number of red blood cells in blood of control, CRS, and different doses (L: 3 mg/kg, M: 10 mg/kg, and H: 30 mg/kg) of CB839-injected CRS mice (n=8 animals) (n=5 animals, one-way ANOVA). (**b**) Number of neutrophils in blood of control, CRS, and different doses (L: 3 mg/kg, M: 10 mg/kg, and H: 30 mg/kg) of CB839-injected CRS mice (n=5 animals, one-way ANOVA). (**c**) Number of lymphocytes in blood of control, CRS, and different doses (L: 3 mg/kg, M: 10 mg/kg, and H: 30 mg/kg) of CB839-injected CRS mice (n=5 animals, one-way ANOVA). (**d**) Number of monocytes in blood of control, CRS, and different doses (L: 3 mg/kg, M: 10 mg/kg, and H: 30 mg/kg) of CB839-injected CRS mice (n=5 animals, one-way ANOVA). (**e**) Number of eosinophils in blood (n=5 animals, one-way ANOVA). (**f**) Number of basophils in blood of control, CRS, and different doses (L: 3 mg/kg, M: 10 mg/kg, and H: 30 mg/kg) of CB839-injected CRS mice (n=5 animals, one-way ANOVA). All data are represented as means ± s.d.. n.s., not significant difference.


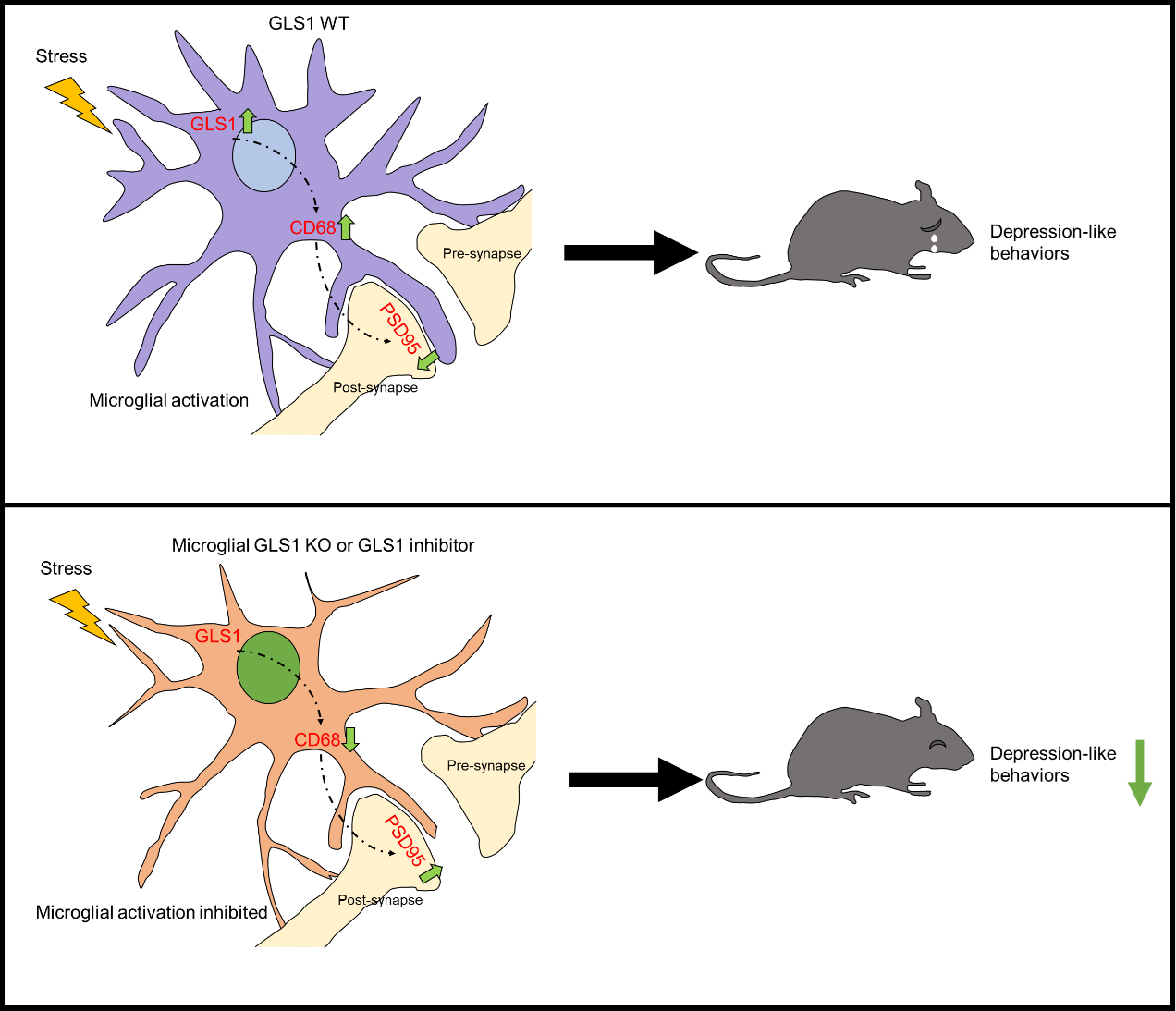


**Supplementary Fig 18. Schematic overview.**

Chronic stress induced mice into depression-like state with higher levels of microglial Gls1 and CD68 expression and further suppressed PSD95 expression. Microglia-specific Gls1 knockout or Gls1 inhibitor reduced the number of activated microglia that possess abnormally heightened phagocytic activities. In this way, microglia-specific Gls1 knockout or Gls1 inhibitor partially rescued synaptic damage and reversed depression-like behaviors after CRS stimulation.

**Reference**

1. Ji, C. et al. Microglial glutaminase 1 deficiency mitigates neuroinflammation associated depression. *Brain Behav Immun*. **99**, 231-245 (2022).
